# Population Structure, Genomic Features, and Antibiotic Resistance of Avian Pathogenic *Escherichia coli* in Shandong Province and Adjacent Regions, China (2008–2023)

**DOI:** 10.3390/microorganisms13071655

**Published:** 2025-07-13

**Authors:** Shikai Song, Yao Wang, Zhihai Liu, Rongling Zhang, Kaiyuan Li, Bin Yin, Zunxiang Yan, Shifa Yang, Shuqian Lin, Yunpeng Yi

**Affiliations:** 1 Shandong Provincial Key Laboratory of Livestock and Poultry Breeding, Institute of Poultry Science, Shandong Academy of Agricultural Science, Jinan 250100, China; sskcau@163.com (S.S.); zhangrongling@saas.ac.cn (R.Z.); 17863801890@163.com (K.L.); yb53650@163.com (B.Y.); yanzunxiang@saas.ac.cn (Z.Y.); yangshifa@saas.ac.cn (S.Y.); shuqianlin@126.com (S.L.); 2 Shandong Animal Disease Prevention and Control Center, Jinan 250100, China; wenshuowy@163.com; 3 College of Chemistry and Pharmaceutical Sciences, Qingdao Agricultural University, Qingdao 266109, China; banyuanjun58@163.com

**Keywords:** avian pathogenic *Escherichia coli*, whole-genome sequencing, population structure, virulence, antimicrobial resistance

## Abstract

Avian pathogenic *Escherichia coli* (APEC) poses a global threat to poultry health and public safety due to its high lethality, limited treatment options, and potential for zoonotic transmission via the food chain. However, long-term genomic surveillance remains limited, especially in countries like China where poultry farming is highly intensive. This study aimed to characterize the population structure, virulence traits, and antimicrobial resistance of 81 APEC isolates from diseased chickens collected over 16 years from Shandong and neighboring provinces in eastern China. The isolates were grouped into seven Clermont phylogroups, with A and B1 being dominant. MLST revealed 27 STs, and serotyping identified 29 O and 16 H antigens, showing high genetic diversity. The minor phylogroups (B2, C, D, E, G) encoded more virulence genes and had higher virulence-plasmid ColV carriage, with enrichment for iron-uptake, protectins, and extraintestinal toxins. In contrast, the dominant phylogroups A and B1 primarily carried adhesin and enterotoxin genes. Antimicrobial resistance was widespread: 76.5% of isolates were multidrug-resistant. The minor phylogroups exhibited higher tetracycline resistance (mediated by *tet*(A)), whereas the major phylogroups showed increased resistance to third- and fourth-generation cephalosporins (due to *bla*_CTX-M_-type ESBL genes). These findings offer crucial data for APEC prevention and control, safeguarding the poultry industry and public health.

## 1. Introduction

*Escherichia coli* (*E. coli*), a pervasively distributed Gram-negative bacterium in the natural environment, normally exists as a commensal inhabitant within the intestinal tracts of humans and animals. Nevertheless, upon the acquisition of specific virulence-associated factors, certain *E. coli* strains can transform into pathogenic variants capable of causing a wide range of diseases. Based on the infection site, pathogenic *E. coli* can be divided into two major categories: intestinal pathogenic *E. coli* (IPEC) and extraintestinal pathogenic *E. coli* (ExPEC) [1]. Avian pathogenic *E. coli* (APEC), which serves as a principal etiological agent of colibacillosis characterized by respiratory, systemic, and reproductive tract infections, represents a prototypical member of the ExPEC group [2].

APEC infects chickens across all age groups, resulting in high mortality, decreased productivity, and considerable economic losses for the global poultry industry [3]. The pathogenic mechanism of APEC is complex, mainly attributed to its encoding of various virulence factors, such as adhesins, toxins, and other effector molecules. Notably, genes like *hlyF*, *ompT*, *iroN*, *iss*, and *iutA* are usually located on a large IncF-type plasmid named ColV [4,5]. These plasmids, with strong horizontal transfer ability, are regarded as markers of hypervirulent APEC strains [6]. Meanwhile, the extensive use of tetracyclines and β-lactam antibiotics in the breeding process has led to a severe APEC antibiotic-resistance issue, especially the emergence of extended-spectrum beta-lactamases (ESBLs), which can confer resistance to most beta-lactam antibiotics, including third- and fourth-generation cephalosporins [7]. The development of APEC resistance has significantly increased the treatment costs of diseased animals.

Beyond animal health, APEC poses a significant public health threat due to its high virulence, rising antimicrobial resistance (AMR), and zoonotic potential via the food chain. Genetically and functionally, APEC closely resembles human ExPEC, particularly uropathogenic *E. coli* (UPEC), sharing key virulence factors and pathogenic mechanisms—highlighting the risk of cross-species transmission [8]. ColV virulence plasmids, common in APEC, are also prevalent in UPEC [9]. Experimental models have shown that APEC can induce urinary tract infection-like symptoms in mice, mirroring human disease [9]. Additionally, APEC frequently harbors mobile resistance genes (e.g., *mcr*, *bla*_CTX-M_), which can disseminate not only through the poultry production chain but also across borders via international trade and migratory wild birds—further compounding the treatment challenges and accelerating the global spread of antimicrobial resistance within the One Health framework [10,11].

To understand the diversity, transmission, and evolutionary characteristics of *E. coli*, and to identify high-risk clones, traditional typing and large-scale genomic analysis are commonly conducted. According to the revised Clermont phylogenetic typing scheme, *E. coli* is divided into eight phylogroups: A, B1, B2, C, D, E, F, and clade I [12]. Recent studies have classified ST117 from group F into group G [13]. The population structure of APEC varies in different reports but mainly includes phylogroups A, B1, B2, and F [14,15]. In terms of multilocus sequence typing (MLST) and serotyping based on surface antigens, strains classified as O antigens O78, O1, and O2, K antigen K1, and sequence types ST117 and ST10 are closely associated with APEC [16]. Evidently, APEC strains with diverse genetic backgrounds can cause avian colibacillosis.

Despite ongoing efforts, critical knowledge gaps remain in our understanding of APEC: (1) long-term genomic surveillance in high-density poultry production areas—particularly in eastern China—has been limited; (2) comparative analyses of virulence and resistance patterns across phylogroups are scarce; and (3) their phylogenetic relationships to global lineages remain poorly characterized. To address these gaps, we performed whole-genome sequencing (WGS) analysis and antimicrobial susceptibility testing on 81 strains isolated from Shandong Province and neighboring regions (Jiangsu, Hebei, Henan) spanning 2008 to 2023. Furthermore, we systematically characterized the population structure of these APEC isolates and compared the inter-population differences in virulence traits and antibiotic resistance profiles. This work clarifies the genetic and phenotypic traits of regional APEC strains, informing future control research, supporting sustainable poultry farming, and aiding public health risk assessment.

## 2. Materials and Methods

### 2.1. Isolation and Identification of APEC Strains

**Figure 1:**
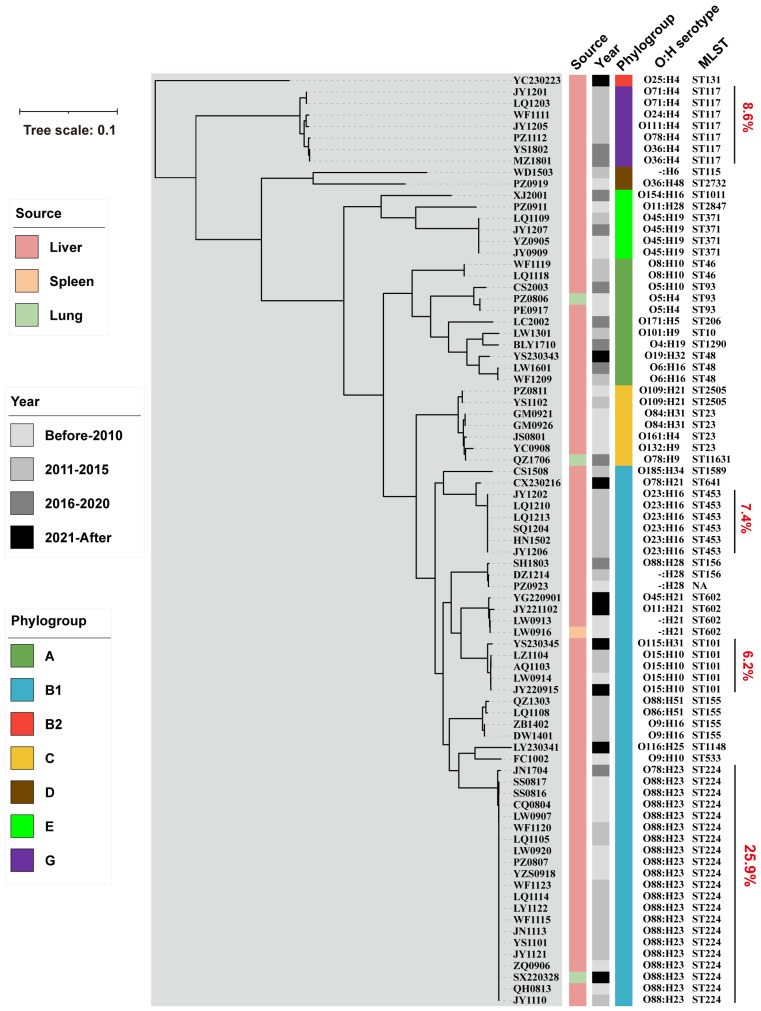
Phylogenetic diversity and population structure of APEC isolates from eastern China. A neighbor-joining (NJ) phylogenetic tree was constructed based on the core-genome SNPs from 81 APEC strains isolated between 2008 and 2023 in Shandong Province and adjacent regions, China. Metadata—including the sample origin (liver, lung, spleen), isolation year, O:H serotypes, multilocus sequence types (STs), and Clermont phylogroups (A, B1, B2, C, D, E, G)—were annotated using iTOL to illustrate the genetic diversity and population structure.

The Poultry Institute, Shandong Academy of Agricultural Sciences (SAAS), intermittently receives sick chicken cases from Shandong and nearby provinces (e.g., Jiangsu, Henan, Hebei) every year. For cases with the typical post-mortem symptoms of extra-intestinal infections, such as perihepatitis, airsacculitis, and fibrinous pericarditis, we collect liver and other representative samples for the isolation of avian pathogenic *Escherichia coli* (APEC). All the sample collection procedures were approved by the Animal Ethics Committee of Shandong Academy of Agricultural Sciences (Approval No. SAAS-2022-32) and conducted in compliance with the guidelines for the ethical treatment of animals. For each sample, the surface was sterilized using a red-hot surgical blade, followed by aseptic collection of internal tissue with an inoculation loop and inoculation onto an antibiotic-free LB plate. Suspected APEC isolates were recovered and identified through 16S ribosomal RNA (rRNA) gene sequencing. In this study, a total of 81 APEC isolates from diseased chickens were recovered between 2008 and 2023, including 25 isolates from 2008 to 2010, 36 from 2011 to 2015, 11 from 2016 to 2020, and 9 from 2021 to 2023. Among these isolates, 77 were derived from liver samples, 3 from lung samples, and 1 from a spleen sample (Figure 1).

### 2.2. Antimicrobial Susceptibility Testing

Fourteen clinically commonly used antibiotics, including ciprofloxacin, ceftriaxone, ceftazidime, cefotaxime, amoxicillin/clavulanic acid, meropenem, tetracycline, tigecycline, trimethoprim/sulfamethoxazole, chloramphenicol, florfenicol, gentamicin, amikacin, and polymyxin, were used as test drugs. The standard strain ATCC 25922 was used as the quality control strain. The minimum inhibitory concentration (MIC) of each antibiotic against the APEC isolates was determined using the internationally standardized microdilution method (CLSI) [17]. Results were deemed reliable only when the MIC values of the quality control strains fell within the specified control ranges; if they fell outside the range, the tests were repeated [18].

### 2.3. Whole-Genome Sequencing

Genomic DNA was extracted from 81 APEC isolates grown in LB broth (37 °C, 180 rpm shaking) to the logarithmic phase (OD_600_ = 0.6–0.8, spectrophotometrically verified) using a HiPure Bacterial DNA Kit (Magen, Guangzhou, China) following the manufacturer’s protocol, and the DNA quality was verified by NanoDrop (Thermo Fisher Scientific, Wilmington, DE, USA; OD260/OD280 = 1.8–2.0; OD260/OD230 > 2). Paired-end DNA libraries for the whole-genome sequencing were prepared using a KAPA HyperPrep Kit (Roche, Basel, Switzerland), which involved end repair, A-tailing, and ligation of Illumina-compatible adapters. Sequencing was conducted on an Illumina NextSeq platform (Illumina, San Diego, CA, USA) using a 2 × 150 bp paired-end configuration, generating approximately 1.0 Gbp of raw data per sample to achieve a target sequencing depth of 50× minimum genome coverage based on the estimated APEC genome size (~5.0 Mbp). Raw reads were processed with FastQC v0.11.9 for quality control, with samples that failed to meet the following criteria excluded from further analysis: average Phred quality score < Q20 across all bases. Trimmomatic v0.39 was used to remove adapter sequences, low-quality bases (Phred quality score < Q20), and short reads (<50 bp) [19]. High-quality reads of each strain were de novo assembled using SPAdes v3.14.1 [20].

### 2.4. Molecular Analysis

The sequence type (ST) of the APEC isolates was determined via SRST2 v0.2.0 by screening the genomic contigs against the PubMLST typing schemes, using the allelic profiles of seven conserved housekeeping genes (*adk*, *fumC*, *gyrB*, *icd*, *mdh*, *purA*, *recA*) [21]. The genotype diversity was evaluated by calculating Simpson’s diversity index with the assistance of BioNumerics (version 7.0; Applied Maths, Sint-Martens-Latem, Belgium). ECTyper v1.0.0 was used to perform the APEC serotype prediction from the assembled genome data [22]. Virulence factors (VFs) and antibiotic resistance genes (ARGs) were detected from the genomic data with Abricate v1.0.1, employing the Ecoli_VF and ResFinder databases, respectively, with thresholds of ≥90% identity and ≥80% coverage [23]. Based on the PointFinder database, ResFinder v4.6.0 with the “--point” parameter was used to detect chromosomal point mutations that contribute to antibiotic resistance [24].

### 2.5. Phylogenetic Analysis and Clermont Phylotyping

Phylogenetic analysis was conducted by constructing an assembly-based alignment of the core-genome single-nucleotide polymorphisms (SNPs) using Parsnp v2.1.4 in the Harvest suite with the default parameters [25]. APEC phylotyping was performed using ClermonTyping 24.02, a computational tool designed for in silico assignment of *Escherichia* strains to phylogenetic groups [26].

### 2.6. Prediction of Virulence-Plasmid ColV

The presence of virulence-plasmid ColV in the APEC strains was predicted based on the WGS data using criteria established by Liu et al. (2018) [5], which require that isolates possess at least four of the following virulence gene clusters: (1) *cvaABC* and *cvi* (the ColV operon); (2) *iroBCDEN* (the salmochelin operon); (3) *iucABCD* and *iutA* (the aerobactin operon); (4) *etsABC* (the putative ABC transporter); (5) *ompT* and *hlyF*; and (6) *sitABCD*.

### 2.7. Data Visualization and Statistical Analysis

Bar charts, pie charts, and violin plots based on the phenotypic and genomic data were generated using GraphPad Prism 8 software. UpSet plots were created by running the UpSetR v1.4.0 package in *R*, following the script described by Li et al. [27]. Heatmaps were generated using TBtools-II version 0.665 [28], and phylogenetic trees were constructed and visualized with iTOL v5 [29]. Adobe Illustrator software CC 2022 was used to standardize the stylistic consistency of the images and generate composite figures. Two-group comparisons were performed in GraphPad Prism 8: Fisher’s exact test for the percentage data, and the non-parametric Mann–Whitney U test for the distributions of the virulence/resistance gene counts, with *p*-values generated for both. Statistical significance was defined as follows: ns (*p* ≥ 0.05), * *p* < 0.05, ** *p* < 0.01, and *** *p* < 0.001.

## 3. Results

### 3.1. APEC Isolates from Eastern China Exhibit a Highly Diverse Population Structure

To investigate the phylogenetic characteristics of APEC strains from Shandong Province and adjacent regions in eastern China, we performed whole-genome sequencing (WGS) on all 81 clinical isolates using the Illumina platform. A neighbor-joining (NJ) phylogenetic tree was constructed from the core-genome allelic profiles, which classified the isolates into seven Clermont phylogroups (A, B1, B2, C, D, E, and G; Figure 1), with A and B1 as the dominant lineages (71.6% combined; Figure S1A). Multilocus sequence typing (MLST) analysis confirmed the high genetic diversity (27 STs, Simpson’s index 91.1%; Table S1), with ST224, ST117, ST453, and ST101 as the most prevalent types (Figure S1B). ST224, ST453, and ST101 belonged to phylogroup B1, while ST117 was classified into phylogroup G (Figure 1). Serotype prediction revealed extensive heterogeneity in the O and H antigens, with O88:H23 as the most common combination (Figure S2A–C). This extensive genetic diversity likely underpins APEC’s adaptability to complex in vivo and in vitro environments. It may facilitate continuous evolution and provide distinct strains with advantages in terms of the virulence, antimicrobial resistance, and other adaptive traits.

### 3.2. APEC Encodes Diverse Extraintestinal Infection-Related Virulence Factors with Marked Differences Across Phylogenetic Groups

**Figure 2:**
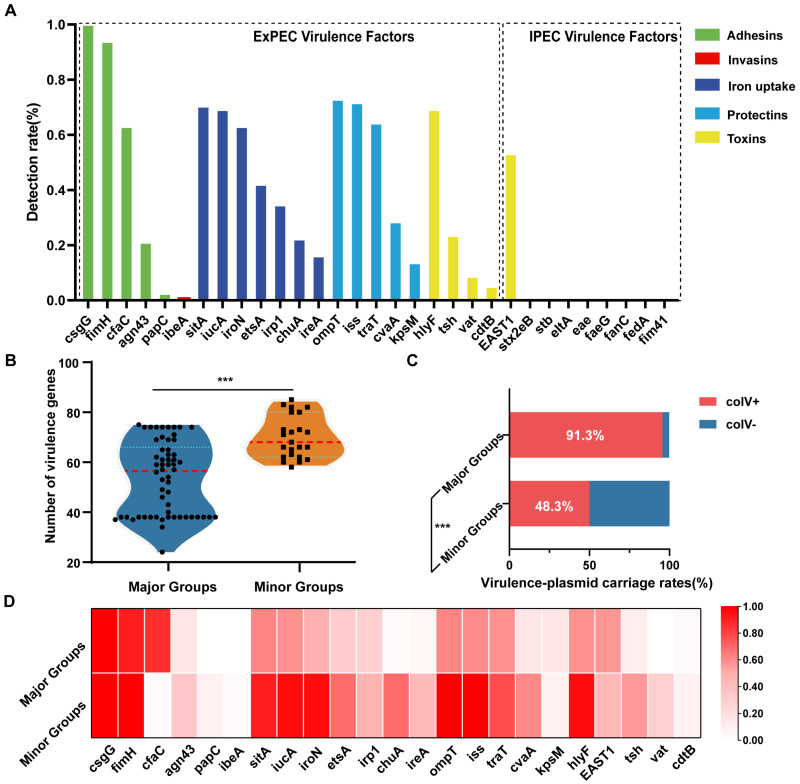
Virulence characteristics and phylogroup-specific differences in 81 APEC isolates. (**A**) Detection rates of five major virulence factor categories—adhesins, invasins, iron-uptake proteins, protectins, and toxins—in 81 APEC strains. These factors were identified through the WGS data and annotated with Abricate using the Ecoli_VF database. The ExPEC Virulence Factors denote those associated with systemic infections in humans and animals, while the IPEC Virulence Factors correspond to genes linked to human diarrheagenic strains. (**B**) Comparison of the number of virulence genes per strain between the major phylogroups (A and B1, *n* = 58) and minor phylogroups (B2, C, D, E, G, *n* = 23). (**C**) Prevalence of the ColV virulence plasmid in the major vs. minor phylogroups. (**D**) Clade-specific differences in the detection rates of key virulence factors, where darker colors indicate higher prevalence. The *p*-values in (**B**) were calculated using the Mann–Whitney U test, and those in (**C**) were determined by Fisher’s exact test (GraphPad Prism 8.0). *** *p* < 0.001.

Using the WGS data, we conducted an in-depth analysis of the APEC virulence factors. In total, 110 virulence-associated genes were identified, encompassing adhesins, invasins, iron-uptake systems, protectins, and toxins (Figure 2A and Figure S3). For the adhesins, type 1 fimbriae (*fim* operon) and curli fimbriae (*csg* operon) were ubiquitous (>90% prevalence) among the isolates. S fimbriae (*cfaC*) and Antigen 43 (*agn43*) were present in 63.0% and 21.0% of strains, respectively, whereas genes for P fimbriae were rare (<5% prevalence). Regarding the iron acquisition systems, genes such as *sitA* (70.3%), *iutA* (69.1%), *iroN* (63.0%), and the *etsABC* operon (42.0%) were highly prevalent. In contrast, genes for yersiniabactin biosynthesis (*irp2*), heme uptake (*chuA*), and iron regulation (*ireA*) had lower prevalence (34.6%, 22.2%, and 16.0%, respectively). In terms of the serum resistance-associated protectins, genes for complement resistance (*traT,* 64.2%) and serum survival (*iss*, 71.6%) were common, as was the colicin V synthesis gene (*cvaC*, 28.4%). Immune-evasion-related genes, including the capsular antigen transporter *kpsM* and outer membrane protease *ompT*, were detected in 72.8% and 13.6% of strains, respectively. For the toxins associated with extraintestinal infections, *hlyF* (encoding α-hemolysin), *tsh* (temperature-sensitive hemagglutinin), *vat* (vacuolating autotransporter toxin), and *cdtB* (cytolethal distending toxin B) were present in 69.1%, 23.5%, 8.6%, and 4.9% of strains, respectively. Among the enterotoxin genes linked to human diarrhea, only the *EAST1* (53.1%) gene was detected. The invasion-associated ibeA gene was rare, being present in only 1.2% of isolates. Additionally, 60.4% (49/81) of the strains were predicted to carry ColV-like virulence plasmids. Replicon typing analysis revealed that 95.9% (47/49) of these plasmids belonged to the Inc*FIB*(AP1918) incompatibility group (Figure S3).

Next, we compared the virulence profiles of strains from the major phylogroups A and B1 (*n* = 58) and the minor phylogroups (B2, C, D, E, G; *n* = 23). Strains from the minor phylogroups carried a higher median number of virulence genes (68.0) than strains from the major phylogroups (56.5; Figure 2B). Likewise, the virulence-plasmid ColV was significantly more prevalent in the minor-clade strains than in the major-clade strains (91.3% vs. 48.3%, *p* = 0.0001, Figure 2C and Figure S3). In terms of the virulence factor categories (Figure 2D), genes related to iron acquisition (*sitABCD*, *iroBCDEN*), protectins (*ompT, iss, cvaA*), and extraintestinal infection-associated toxins (*hlyF, tsh, vat, cdtB*) were more abundant (*p* < 0.05) in the minor phylogroups, whereas genes encoding adhesin-associated fimbriae (*cfaC,* 86.2% vs. 4.3%, *p* < 0.0001) and enteric disease-associated toxins (*EAST1*, 56.9% vs. 43.5%, *p* = 0.3281) were more prevalent in the major phylogroups. These findings indicate distinct differences in the virulence characteristics between strains from different phylogroups, which may reflect divergence in their survival strategies and infection preferences within hosts.

### 3.3. APEC Strains Exhibit High Prevalence of Antibiotic Resistance

**Figure 3:**
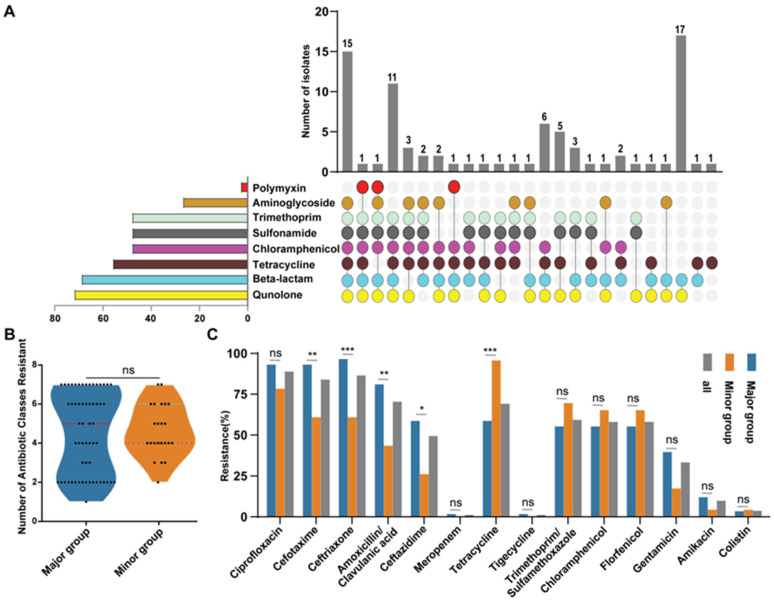
Antibiotic resistance profiles of APEC isolates (*n* = 81) from chickens with colibacillosis in eastern China over the 2008–2023 study period. (**A**) UpSet plots reflecting the multidrug resistance patterns of the APEC strains against 8 antibiotic classes. In the central dot matrix, each column represents a unique resistance phenotype profile. The colored dots within the column indicate resistance to at least one antimicrobial agent in the corresponding drug class, with the colors matching specific drug categories. The upper histogram shows the total number of strains with a specific resistance phenotype profile, while the left histogram displays the number of strains resistant to each drug class. Sulfamethoxazole/trimethoprim, a combination antibiotic, was classified under both the sulfonamides and trimethoprim categories for statistical analysis in this study. (**B**) Comparison of the number of antibiotic classes to which strains from the major phylogroups (A and B1, *n* = 58) and minor phylogroups (B2, C, D, E, G, *n* = 23) were resistant. (**C**) The prevalence of antimicrobial resistance in four common STs of ExPEC. The *p*-values in (**B**) were calculated using the Mann–Whitney U test, and those in (**C**) were determined by Fisher’s exact test (GraphPad Prism 8.0). ns, no significance; * *p* < 0.05, ** *p* < 0.01, *** *p* < 0.0001.

We assessed the antibiotic susceptibility of the APEC isolates to 14 antibiotics spanning eight classes. All the isolates were resistant to at least one antibiotic class, and the overall multidrug resistance (MDR; defined as resistance to antibiotics from ≥3 classes) rate exceeded 76.5% (62/81; Figure 3A). The resistance rates varied substantially across the antibiotic classes; the highest resistance was observed for fluoroquinolones (88.9%, 72/81) and β-lactams (85.2%, 69/81), whereas the lowest was observed for polymyxins (3.7%, 3/81). The resistance rates concerning tetracyclines, chloramphenicol, trimethoprim/sulfamethoxazole, and aminoglycosides were 69.1% (56/81), 59.3% (48/81), 59.3% (48/81), and 33.3% (27/81), respectively.

We then compared the antibiotic resistance patterns between the major- and minor-clade strains. There was no significant difference in the median number of antibiotic classes to which strains from the two groups were resistant (5.0 vs. 4.0, *p* = 0.9576, Figure 3B). Analysis of individual antibiotics showed that most resistance rates were similar between the phylogroups. Both groups exhibited high resistance to ciprofloxacin (93.1% vs. 78.3%, *p* = 0.1091), trimethoprim/sulfamethoxazole (55.2% vs. 69.6%, *p* = 0.3147), chloramphenicol (55.2% vs. 65.2%, *p* = 0.4621), and florfenicol (55.2% vs. 65.2%, *p* = 0.4621); moderate resistance to gentamicin (39.6% vs. 17.4%, *p* = 0.0696); and relative sensitivity to amikacin (12.1% vs. 4.3%, *p* = 0.4293), meropenem (1.7% vs. 0%, *p* = 0.9999), tigecycline (1.7% vs. 0%, *p* = 0.9999), and colistin (3.4% vs. 4.3%, *p* = 0.9999; Figure 3C). Notable exceptions included the significantly higher resistance in the major-clade strains to ceftriaxone (96.6% vs. 60.9%, *p* < 0.0001), ceftazidime (58.6% vs. 26.1%, *p* = 0.013), cefotaxime (93.1% vs. 60.9%, *p* = 0.0011), and amoxicillin/clavulanic acid (81.0% vs. 43.5%, *p* = 0.0023). Conversely, the minor-clade strains exhibited significantly higher resistance to tetracycline (95.7% vs. 58.6%, *p* = 0.0010; Figure 3C).

### 3.4. APEC Genomes Harbor Diverse Antibiotic Resistance Genes

**Figure 4:**
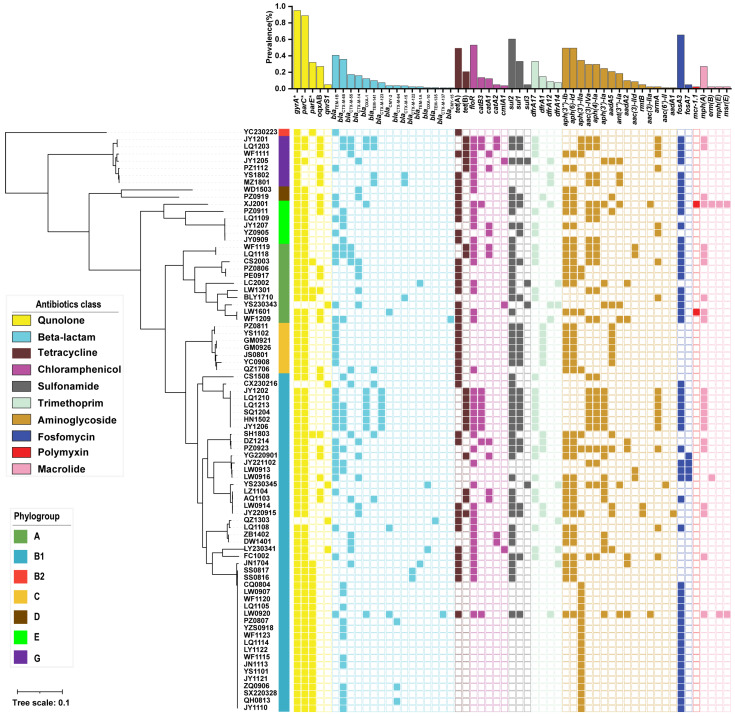
Distribution of the antibiotic resistance determinants across 81 APEC genomes. The central heatmap displays the presence (filled squares) or absence of 54 antibiotic resistance genes and three chromosomal resistance mutations, organized into ten antibiotic-class groups (columns color-coded by class). Above the heatmap, a bar chart quantifies the number of isolates carrying each determinant. To the right, a core-genome SNP phylogeny annotated with the seven Clermont phylogroups links each strain’s lineage to its resistance profile. The asterisk (*) indicates that the gene has undergone at least one point mutation associated with antibiotic resistance.

To investigate the genetic determinants of antibiotic resistance, we screened all the sequenced genomes for antibiotic resistance genes (ARGs) and for point mutations in three chromosomal genes (*gyrA*, *parC*, and *parE*). In total, we identified 54 ARGs and point mutations potentially conferring resistance to 10 antibiotic classes (Figure 4). Notably, every genome harbored four or more ARGs. For fluoroquinolone, high-frequency mutations were found in *gyrA* (96.3%, 78/81), *parC* (90.1%, 73/81), and *parE* (32.1%, 26/81). Two fluoroquinolone resistance genes, *oqxAB* and *qnrS1*, were also detected in 27.2% (22/81) and 4.1% (5/81) of the strains, respectively. β-lactamase genes were the most abundant resistance determinants. We detected 16 β-lactamase gene types across the 81 strains, with *bla*_TEM-1B_ being the most prevalent (40.7%, 33/81). Notably, nine extended-spectrum β-lactamase (ESBL) genes conferring resistance to third- and fourth-generation cephalosporins were identified in 75.3% (61/81) of the isolates. The individual ESBL genes with prevalence above 5% included *bla*_CTX-M-65_ (35.8%, 29/81), *bla*_CTX-M-55_ (17.3%, 14/81), *bla*_CTX-M-14_ (16.0%, 13/81), and *bla*_CTX-M-123_ (7.4%, 6/81). The tetracycline resistance genes *tet*(A) and *tet*(B) were detected in 49.4% (40/81) and 21.0% (17/81) of the strains, respectively. Among the five florfenicol resistance genes, *floR* was the most prevalent (53.1%, 43/81), followed by *catB3* (13.6%, 11/81) and *catA1* (12.3%, 10/81); the remaining florfenicol genes each occurred in fewer than 5% of strains. The sulfonamide resistance gene *sul2* was encoded by 60.5% (49/81) of isolates. Of the 15 aminoglycoside resistance genes detected, 9 were present in more than 5% of strains, with *aph(3″)-Ib* and *aph(6)-Id* being the most common (both 49.4%, 40/81). The colistin resistance gene *mcr-1.1* was identified in 2.5% (2/81) of isolates. In addition, two fosfomycin resistance genes and four macrolide resistance genes were detected among the sequenced strains.

**Figure 5:**
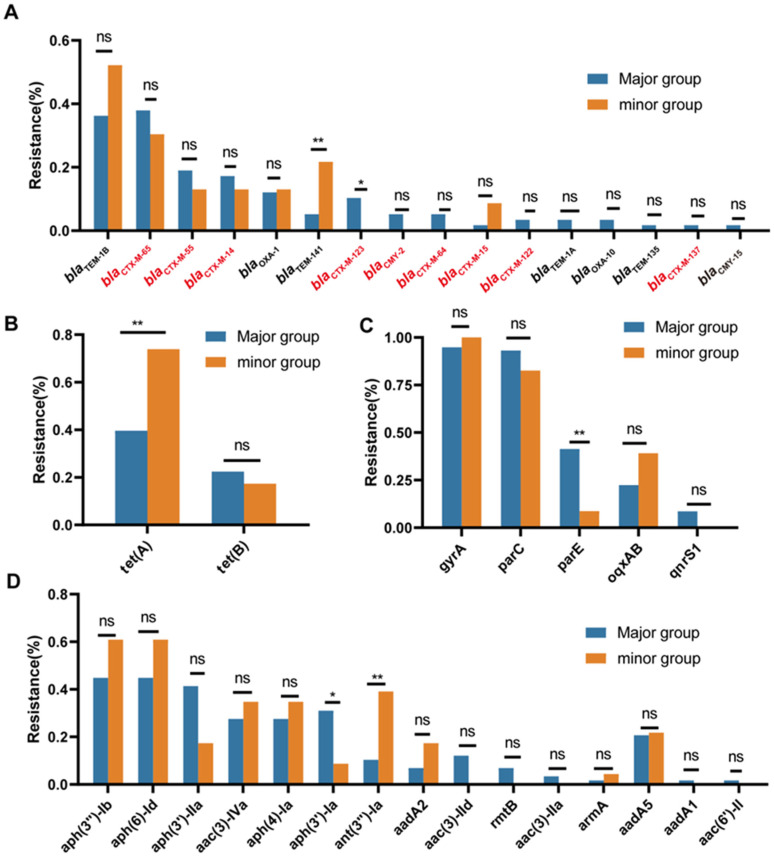
Comparison of the resistance gene detection rates for β-lactams (**A**), tetracyclines (**B**), fluoroquinolones (**C**), and aminoglycosides (**D**) across the APEC phylogroups. Notes: The major phylogroups include A and B1 (*n* = 58), while the minor phylogroups include B2, C, D, E, and G (*n* = 23). Extended-spectrum β-lactamase (ESBL)-encoding resistance genes are shown in the red font. the *p*-values in (**A**–**D**) were determined by Fisher’s exact test (GraphPad Prism 8.0). ns, no significance; * *p* < 0.05, ** *p* < 0.01.

Differences in the repertoire and/or prevalence of β-lactam and tetracycline resistance genes were observed between the major and minor phylogroups, potentially underlying their divergent antibiotic resistance profiles. For example, the ESBL-encoding genes *bla*_CTX-M-123_, *bla*_CMY-2_, *bla*_CTX-M-64_, *bla*_CTX-M-122_, and *bla*_CTX-M-137_ were exclusively detected in the major phylogroups (Figure 5A). Conversely, *tet*(A) showed significantly higher prevalence in the minor phylogroups compared to the major phylogroups (73.9% vs. 39.7%, *p* = 0.0069), corroborating the results from the antibiotic susceptibility testing (Figure 5B). In addition, although the detection rates of certain resistance genes differed between the main and minor phylogroups, complementary changes in the resistance genes within the same functional category may have counteracted the effects of single genes, leading to no significant differences in the resistance phenotypes between the two groups. For instance, *parE* mutations associated with fluoroquinolone resistance were detected at higher rates in the main clade, while *oqxAB* showed lower detection rates in the main clade compared to the minor clade (Figure 5C). Regarding aminoglycoside resistance, the *aph(3′)-Ia* gene had a higher detection rate in the main clade, whereas *ant(3″)-Ia* was more prevalent in the minor clade (Figure 5D).

## 4. Discussion

APEC is the primary pathogen causing avian colibacillosis, with its high mortality rate and decline in production performance imposing significant economic losses on the global poultry industry. Epidemiological surveillance reveals notable regional heterogeneity in APEC infection rates (9.52–36.73%) [2]. As the world’s largest poultry producer, China’s epidemiological characteristics and antimicrobial resistance evolution trends in terms of APEC remain incompletely elucidated. To address this, this study focused on core breeding regions in eastern China (Shandong and surrounding provinces), isolating 81 APEC strains from typical cases between 2008 and 2023. Through whole-genome sequencing combined with phenomics analysis, we systematically characterized their genetic evolution, virulence traits, and resistance transmission risks.

Combined analysis of the core-genome SNP profiles and Clermont typing showed that 71.6% (58/81) of the strains belonged to phylogenetic groups A (13.6%) and B1 (58.0%), significantly differing from the traditional B2/D group dominance in extraintestinal pathogenic *E. coli* (ExPEC) [14]. Notably, groups A/B1 are generally considered dominant commensal populations in avian intestines [30,31]. Their high prevalence suggests APEC may not represent an independent evolutionary lineage but rather evolve from commensal bacteria through horizontal acquisition of virulence genes. Similarly, Li et al. reported analogous conclusions in porcine ExPEC, where over 80% of Chinese pig-derived ExPEC isolates were classified into commensal-associated groups A and B1, implying their origin from porcine intestinal microbiota [27]. This inference aligns closely with the virulence factor distribution: the A/B1 strains exhibited higher detection rates of the adhesion-related gene *cfaC* (63.0%) and enterotoxin gene *EAST1* (53.1%) than the other groups. Adhesion factors confer colonization advantages on intestinal mucosa [32], while *EAST1* may disrupt intestinal barriers via local inflammation induction, facilitating commensal translocation to extraintestinal tissues [33]. This process of intestinal colonization followed by extraintestinal tissue translocation, cooperatively mediated by adhesion factors and enterotoxin *EAST1*, likely serves as a key strategy for APEC to breach the host’s immune defenses.

ColV-like virulence plasmids are key mediators of APEC’s ability to invade extraintestinal sites and cause systemic infection [34]. These large IncF-family plasmids encode iron acquisition systems (*sitABCD*, *iroBCDEN*) and immune evasion factors such as iss, enhancing bacterial survival in the iron-limited, complement-rich bloodstream [35]. Their high conjugative transfer efficiency facilitates rapid dissemination across *E. coli* populations [6]. Importantly, ColV plasmids are not unique to APEC; they are also prevalent in human ExPEC, particularly UPEC, where ColV-encoded toxins (*hlyF*, *tsh*) play key roles in pathogenesis [8,9]. In our dataset, ColV carriage was nearly universal in the classical extraintestinal phylogroups (B2, C, D, E, G; 91.3%) and was also detected in 48.3% of the strains from the commensal-associated groups A and B1. This broad phylogenetic distribution suggests that horizontal transfer of ColV can erode the genomic distinctions between commensal and pathogenic *E. coli*. Moreover, the enrichment of the extraintestinal toxins (*hlyF*, *tsh*) in the minor phylogroups further amplifies concerns about the convergence of virulence factors between APEC and human ExPEC, hinting at potential cross-species transmission and shared evolutionary pathways.

The MLST typing in this study revealed that the 81 APEC isolates exhibited high genetic diversity, yielding 27 distinct sequence types (STs). The dominant clones ST224 (25.9%) and ST117 (8.6%) matched international high-risk clones previously identified in multiple countries (China, Brazil, Hungary) and across diverse hosts, including broilers, wild birds, and humans [36,37]. This suggests that the transnational poultry trade and wild bird migration may accelerate the global dissemination of APEC clones [38].

More critically, the APEC isolates in this study exhibited consistently high antimicrobial resistance levels, as previously reported [15,39], posing severe challenges for infection control: 76.5% of strains showed multidrug resistance (MDR), with the resistance rates in relation to commonly used antibiotics such as fluoroquinolones (88.9%), β-lactams (85.2%), tetracyclines (69.1%), chloramphenicol (59.3%), and trimethoprim–sulfamethoxazole (59.3%) significantly exceeding expectations. Molecular analysis showed that 75.3% of isolates carried extended-spectrum β-lactamase (ESBL) genes, mainly *bla*_CTX-M-65_, *bla*_CTX-M-55_, and *bla*_CTX-M-14_. These variants are increasingly reported in meat products, wastewater, and clinical *E. coli* isolates, suggesting a high potential for foodborne and environmental transmission [40]. Such findings underscore the One Health significance of APEC as both a pathogen and a reservoir of AMR genes that may spill over into human populations.

## 5. Limitations

This study has several limitations. First, all the APEC isolates were obtained from clinically diseased chickens, potentially introducing bias toward highly pathogenic strains and underrepresenting asymptomatic or mildly virulent lineages. Second, although the isolates span a 16-year period, the annual sampling was limited and uneven, constraining the resolution of temporal evolutionary trends. Third, this study focused solely on avian clinical isolates, lacking samples from the environment, food chain, or humans. This hinders comprehensive assessment of cross-species transmission and limits interpretation within a One Health framework. Fourth, while we observed that the A/B1 strains exhibited higher resistance to tetracyclines and sulfonamides, and isolates from phylogroups B2, C, D, and G showed increased resistance to third-generation cephalosporins, we were unable to provide a robust explanation for these resistance profile differences. Additionally, we found no existing literature that adequately accounts for this phenomenon. These disparities may potentially be linked to the variable antibiotic exposure across distinct ecological niches or differential compatibility between resistance plasmids and specific chromosomal backgrounds, though this remains speculative. Future research should incorporate systematic, longitudinal, and multi-host sampling to better capture the full ecological and evolutionary landscape of APEC.

## 6. Conclusions

In summary, our 16-year study underscores that APEC remains a dynamic and evolving pathogen in eastern China. The prolific diversity, pervasive virulence traits, and alarming resistance profiles we observed call for sustained genomic surveillance and coordinated control efforts. These findings reinforce the zoonotic potential of APEC and its relevance to the One Health framework, underscoring the urgent need for integrated surveillance and responsible antibiotic use in animal agriculture.

## Data Availability

The whole-genome sequence data of the 81 APEC strains have been submitted to the NCBI under the BioProject accession number PRJNA1264773.

## Supplementary Materials

The following supporting information can be downloaded at: https://www.mdpi.com/article/10.3390/microorganisms13071655/s1, Figure S1: The population structure of 81 APEC strains from Shandong Province and adjacent regions in China; Figure S2: Serotype prediction of 81 APEC strains from Shandong and adjacent Chinese regions using ECTyper v1.0.0 based on WGS data; Figure S3: Phylogenetic tree of 81 APEC strains integrating virulence genes and ColV virulence plasmid types; Table S1: Simpson’s diversity index analysis of APEC isolates from chickens with colibacillosis in eastern China over the 2008–2023 study period.
