# Supplementary material for: Population Structure, Genomic Features, and Antibiotic Resistance of Avian Pathogenic Escherichia coli in Shandong Province and Adjacent Regions, China (2008–2023)
Source: Microorganisms. 2025 Jul 13;13(7):1655. doi: 10.3390/microorganisms13071655 (PMC12298790; doi:10.3390/microorganisms13071655)
Supplement: Supplementary file 1 [file microorganisms-13-01655-s001.zip › microorganisms-3689932-supplementary.pdf]

Supporting Information for

Population Structure, Genomic Features, and Antibiotic Resistance of Avian Pathogenic *Escherichia coli* in Shandong Province and Adjacent Regions, China (2008-2023)

Shikai Song[1], Yao Wang[2], Rongling Zhang[1], Kaiyuan Li[1], Zhihai Liu[3], Bin Yin[1], Zunxiang Yan[1],Shifa Yang[1], Zengcheng Zhao[1], Yunpeng Yi[1*], Shuqian Lin[1*]

[1]Poultry Research Institute, Shandong Academy of Agricultural Science, Jinan, Shandong 250100, China; sskcau@163.com(S.S.); zhangrongling@saas.ac.cn(R.Z); 17863801890@163.com(K.L.); yb53650@163.com(B.Y.); 743318367@qq.com(Z.Y.); yangshifa@saas.ac.cn(S.Y.); zhaozeng-cheng@sina.com(Z.Z.); yiyp@foxmail.com(Y.Y.); shuqianlin@126.com(S.L.)
[2]Shandong Animal Disease Prevention and Control Center, Jinan, Shandong 250100, China; wenshuowy@163.com(Y.W.)
[3]College of Chemistry and Pharmaceutical Sciences, Qingdao Agricultural University, Qingdao, Shandong 266109, China; banyuanjun58@163.com(Z.L.)
*Correspondence: yiyp@foxmail.com(Y.Y.); shuqianlin@126.com(S.L.)

This PDF file includes:

Figure S1. The population structure of 81 APEC strains from Shandong Province and adjacent regions in China.

Figure S2. Serotype prediction of 81 APEC strains from Shandong and adjacent Chinese regions using ECTyper v1.0.0 based on WGS data.

Table S1. Simpson's diversity index analysis of APEC isolates from from chickens with colibacillosis in eastern China over the 2008-2023 study period.

Figure S3. Phylogenetic tree of 81 APEC strains integrating virulence genes and ColV virulence plasmid types.

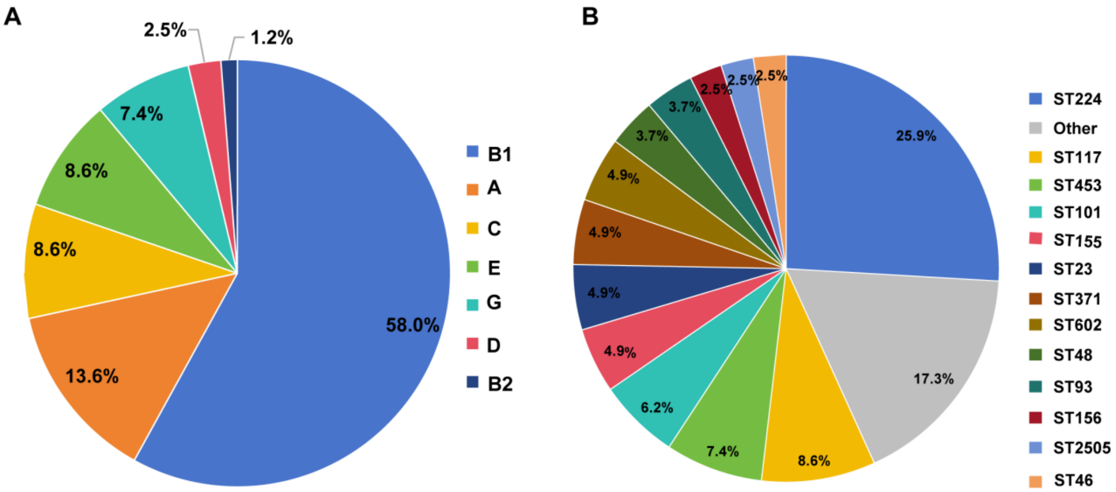

Figure S1. The population structure of 81 APEC strains from Shandong Province and adjacent regions in China. (A) According to the revised Clermont phylogenetic typing scheme, APEC were classified into seven phylogroups: A, B1, B2, C, D, E, and G. (B) Sequence type (ST) analysis of 81 APEC strains according to PubMLST typing schemes. Note: ST types with fewer than 1 isolate were included in “other”.

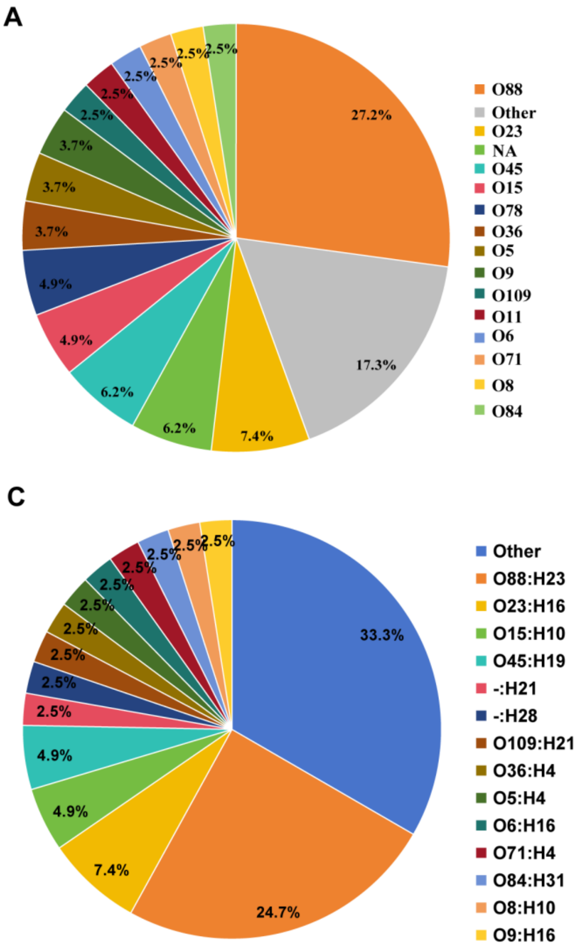

Figure S2. Serotype prediction of 81 APEC strains from Shandong and adjacent Chinese regions using ECTyper v1.0.0 based on WGS data. (A) O-antigen serotype prediction via analysis of O-antigen-related gene clusters (*wzx*, *wzy*, *wzm*, *wzt*). (B) H-antigen serotype prediction via analysis of *fliC*, *flkA*, *fllA*, *flmA* and *flnA* Genes. (C) Combined O-H serotype generation from predicted O and H serotypes. Note: Serotypes with fewer than 1 isolate are individually displayed and classified into the "other" category. Both "NA" and "-" denote failed serotype prediction.

Table S1. Simpson's diversity index analysis of APEC isolates from from chickens with colibacillosis in eastern China over the 2008-2023 study period.

	number of isolates	number of genotypes (ST)	Simpson's diversity index
All	81	27	91.1%
Major phylogroups	58	17	84.6%
Minor phylogroups	23	10	86.6%

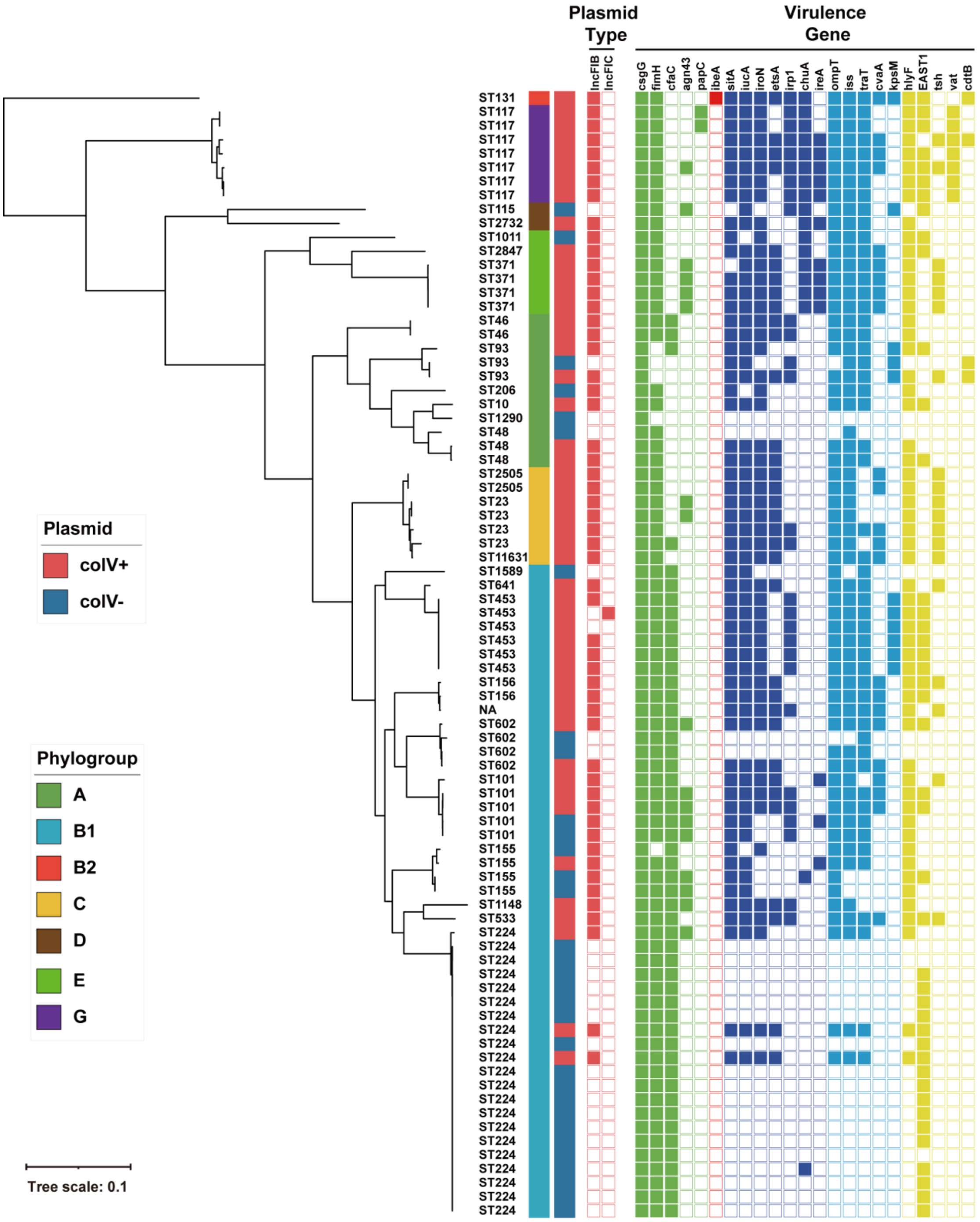

Figure S3. Phylogenetic tree of 81 APEC strains integrating virulence genes and ColV virulence plasmid types. Note: colV+ and colV- respectively denote that each strain was predicted to carry or not carry the virulence plasmid ColV according to the criteria defined by Liu et al[1]. Plasmid type refers to the replicon type of the ColV plasmid identified by the plasmidFinder software.

References

1. C. M. Liu, *et al.*, Escherichia coli ST131-H22 as a Foodborne Uropathogen. *MBio* **9** (2018).
